# Smartphone Authentication System Using Personal Gaits and a Deep Learning Model

**DOI:** 10.3390/s23146395

**Published:** 2023-07-14

**Authors:** Jiwoo Choi, Sangil Choi, Taewon Kang

**Affiliations:** Department of Computer Science and Engineering, Gangneung-Wonju National University, Wonju 26403, Republic of Korea; cephular@gmail.com (J.C.); twkang@gwnu.ac.kr (T.K.)

**Keywords:** human gait, convolutional neural network, authentication, machine learning

## Abstract

In a society centered on hyper-connectivity, information sharing is crucial, but it must be ensured that each piece of information is viewed only by legitimate users; for this purpose, the medium that connects information and users must be able to identify illegal users. In this paper, we propose a smartphone authentication system based on human gait, breaking away from the traditional authentication method of using the smartphone as the medium. After learning human gait features with a convolutional neural network deep learning model, it is mounted on a smartphone to determine whether the user is a legitimate user by walking for 1.8 s while carrying the smartphone. The accuracy, precision, recall, and F1-score were measured as evaluation indicators of the proposed model. These measures all achieved an average of at least 90%. The analysis results show that the proposed system has high reliability. Therefore, this study demonstrates the possibility of using human gait as a new user authentication method. In addition, compared to our previous studies, the gait data collection time for user authentication of the proposed model was reduced from 7 to 1.8 s. This reduction signifies an approximately four-fold performance enhancement through the implementation of filtering techniques and confirms that gait data collected over a short period of time can be used for user authentication.

## 1. Introduction

We live in a hyper-connected society where humans and machines, and even machines alone, connect to exchange and generate information. This is due to the development of wired and wireless communication technology, the Internet, big data production and consumption, and artificial intelligence. With the development of humanoid robots with human learning capabilities, machines are expected to perform increasingly many tasks and provide optimal solutions to humans. However, this convenience comes with the need to share personal information, which can lead to security concerns.

One of the most popular ways to protect personal information is password authentication; however, there is a risk that passwords can be forgotten, lost, or stolen by malicious users [1]. The use of human biometric data, such as fingerprint, iris, and face recognition, is a more convenient and safer method. In this study, we examined whether human walking could be introduced as a new biometric authentication method, which has not been accomplished before, using a smartphone.

Previous gait studies on user authentication do not reflect the real-world environment by fixing the sensor’s position on the body or collecting data in a limited experimental environment such as a flat surface or treadmill [2]. In real-world environments, gait features can change due to sensor position and walking environment [3], which can lead to misidentification in authentication systems based on limited environments. In this study, gait data were collected from various positions on the body in a near real-world experimental setting, and data from all positions were trained to the identification model as one training set, not separated by position. Thus, the smartphone user authentication system proposed in this paper can be easily applied in real life. The smartphone user authentication system uses a convolutional neural network (CNN) deep learning model trained on legitimate user gait data and then enters the gait data for authentication into the model to perform user authentication. This allows legitimate users to use their device while preventing illegal users.

Our contributions are summarized as follows:

1. We train the authentication system with gait data, including data collected not only from one position on the body but also from other positions, so that it can be easily applied to real-world environments where gait data can be collected from various positions on the body;
2. The system proposed in this study showed an authentication accuracy of more than 90% even though the time required for authentication was as short as 1.8 s. This shows that even short periods of gait data can be utilized for user authentication.

The remainder of this paper is structured as follows. Section 2 examines related research trends in user authentication methods based on gait analysis. Section 3 describes the proposed authentication system in detail. In Section 4, deep learning models are evaluated. Finally, Section 5 presents a conclusion and discusses future research tasks.

## 2. Related Work

Gait research is being conducted in various fields, such as medicine, healthcare, and security. Gait identification, which involves identifying an individual through a unique gait pattern [4,5,6,7,8], has been actively studied, and authentication models and systems based on gait patterns have been proposed as new security authentication mechanisms. However, most studies aimed at authentication or gait identification collect or use open datasets with limited collection environments, such as RecodGait, OU-ISIR, WhuGAIT, ZJU-GaitAcc, Motion Sensor, WISDM and HMOG, which have limitations, shown in Table 1, due to the collection environment of the gait data.

**Table 1:** Limitations of the literature in gait data collection.

Literature Opendata [Citation #]	Collect Position for System Training	Collection Environment	Limitation
RecodGait [9]	Fixed: pocket	Dynamic: outdoor	(a)
OU-ISIR [10,11,12,13]	Fixed: back waist	Controlled: treadmill	(a), (b)
ZJU-GaitAcc [14]	Fixed ^: wrist, upper arm, pelvis, thigh, ankle	N/A	(a)
Motion Sensor [15]	Fixed: pocket	Dynamic: multiple activities (stand, walk, up/downstairs, jog, and sit)	(a)
WISDM [9,12,15]	Fixed: pocket	Dynamic: multiple activities	(a)
HMOG [12,16]	Fixed: hand	Dynamic: walk, read, and write	(a)
[17,18,19,20]	Fixed ^: arm, wrist, waist, thigh, pocket …	Dynamic: multiple activities, Indoor, Outdoor	(a)
[2,21,22]	Fixed: thigh, pocket, wrist	Controlled: walking direction, plain surface	(a), (b)

^ Data collected from multiple positions but only trained and tested in one position. (a) An authentication system trained on data from one position may misclassify data from another position. (b) An authentication system trained on data from controlled environments may misclassify data from uncontrolled environments.

The open datasets in Table 1, OU-ISIR and HMOG, contain data collected only from treadmills or with fixed sensor positions. In addition to studies using open datasets, some have collected gait data on flat surfaces or with fixed sensor positions. In particular, all the studies shown in Table 1 only train the authentication system on gait data collected at a fixed sensor position, which does not reflect the real-world environment where the data collection position changes frequently. These studies clarified that the environment in which gait data are collected needs to be considered.

The models used for authentication and identification after gait data collection vary; however, CNNs [10,12,13,15,20,21,22,23] and long short-term memory (LSTM) [12,24] have exhibited good performance in many studies. Others include the support vector machine (SVM) [13,17,18,25], K-nearest neighbors (KNN) [17,26], and random forest (RF) [18,26].

A limitation of previous studies is that they used data collected from only one position on the body to train the system, which does not reflect the real world. In this study, gait data were collected from various positions on the body to reflect real-world environments as accurately as possible. This study aimed to evaluate the results of the proposed smartphone authentication system and verify that it can be utilized as a new secure authentication method.

## 3. Data Collection and Preprocessing

Section 3 describes the process of collecting gait data and three preprocessing steps to improve the performance of the system. Ten individuals [17,21,24,26] participated in this study. Acceleration and angular velocity data were collected from inertial sensors embedded in their smartphones. Data collected in this manner are called gait data.

**Figure 1:**
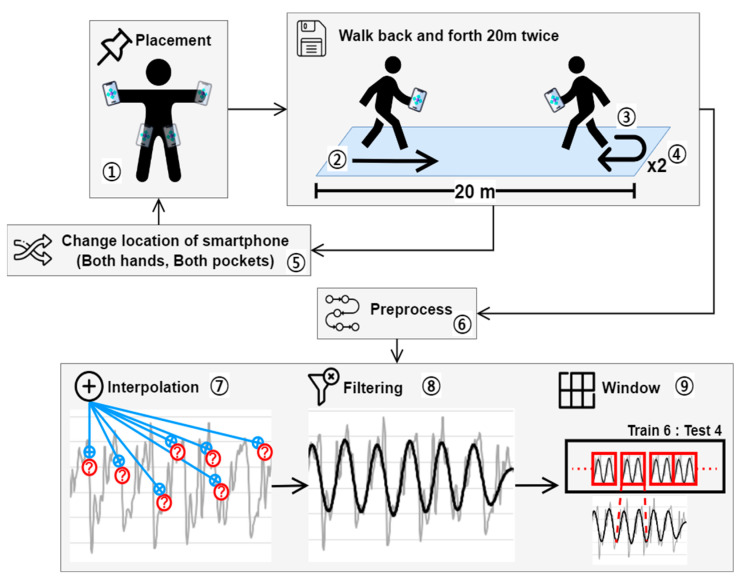
Overview of gait data collection and preprocessing.

**Figure 2:**
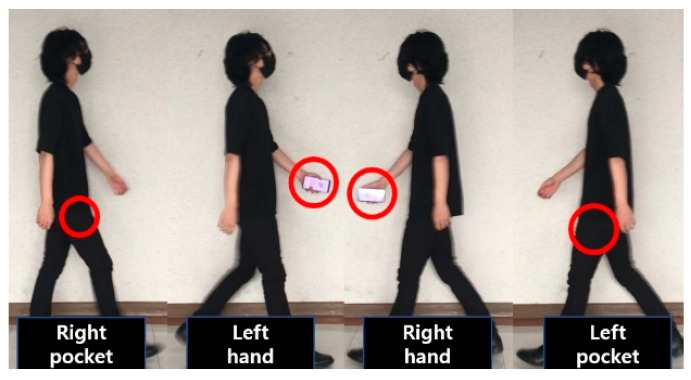
Gait data collection at different places.

Let us look at Figure 1, which outlines Section 3. Gait data were collected by placing smartphones in both hands and both pants pockets, as shown in Figure 1 and Figure 2, and participants were instructed to walk back and forth across a flat surface of approximately 20 m twice while maintaining a natural speed. Our study does not separate the data into 20-m walk segments. We collect data continuously from the time the smartphone is placed at the first position on the body until it is placed at the fourth position and walks 20 m back and forth, thus collecting only one raw dataset. This dataset is then preprocessed, and the authentication system uses data from all positions on the body for training, not just one position. Therefore, the data collected can be said to be from multiple positions on the body. The size of the collected gait data for each participant is shown in Table 2, separated by the position of the smartphone and the average time taken to measure per person (ID), as shown in Figure 2. It took one person an average of 94.3 s to collect data from all four positions, with an average gait data size of 1.46 MB. Before conducting the experiment, all participants completed a personal information consent form for the use of their gait data and were aware of the precautions related to the experiment. The Institutional Review Board (IRB) of Gangneung-Wonju National University (GWNUIRB-2020-33) granted us permission to conduct our experiment. An LG G8 ThinQ (LM-G850N) smartphone was used to collect the gait data at a sampling rate of 50 Hz (20 ms).

**Figure 3:**
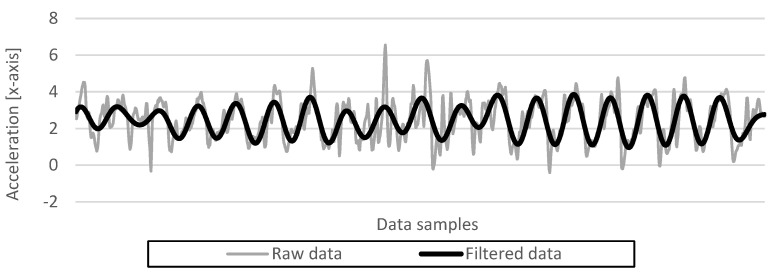
Acceleration after low−pass filtering.

Three preprocessing tasks shown in Figure 1 were conducted before using the collected gait data: linear interpolation to supplement missing data, noise filtering to extract only gait features from the data, and window processing to organize the individual data collected during each sampling period into one continuous dataset. Linear interpolation compensates for missing data with an error of 0.5 to 1 ms, which should be stored in a 20-ms sampling period using Equation (1) below. Filtering removes noise, such as smartphone shaking, from the gait data to extract only gait features; Figure 3 shows the acceleration before and after filtering as gray and black solid lines, respectively. Let us compare these lines. The solid gray line has an irregular amplitude or period, making it difficult to find a specific pattern, whereas the solid black line has a constant amplitude and period with a regular and repetitive pattern. The low-pass filter from the Python-based Butterworth library was used to filter both acceleration and angular velocity data. Filtering with a short period of 20 ms is highly resource-consuming for the hardware, so filtering with a short period may cause delays in the system on low-performance mobile devices. Therefore, we adjusted the Python filtering parameters to extend the filtering period to reduce the burden on the hardware and applied filtering with a period of 100 ms, which is the optimal performance for the proposed authentication system.

(1) $$y=y_{0}+\left(x-x_{0}\right)\frac{y_{1}-y_{0}}{x_{1}-x_{0}},\;x:target\;time;\;y:target\;value;\;x_{0},x_{1}:time\;before\;and\;after\;x,\;respectively;\;y_{0},y_{1}:value\;before\;and\;after\;x,\;respectively.$$

After filtering, as previously mentioned, window processing organizes the individual data collected during each sampling period into a continuous dataset for a certain amount of time. Owing to the repetitive and continuous characteristics of walking, as shown in Figure 3, when analyzing gait data, it may be more effective to find an individual’s gait pattern by combining data from a certain period into a single dataset rather than treating each point in time individually. In this study, the length of the dataset was set to 1.8 s to perform window processing. The length of the dataset was the optimal length obtained by monitoring the performance of the system, and the raw dataset collected over 1.8 s is called a window. Consider Equation (2), which shows the composition of the window. A window is defined as a set of inertial data generated over 1.8 s, consisting of 100 ms intervals and a total of 18 inertial data. This window dataset is split 6:4 to serve as the training and test datasets for the authentication model described in Section 4.

(2) $${Window}_{i}=\left|\begin{array}{cc} M_{{acc}_{i}} & M_{{gyro}_{i}}\\ M_{{acc}_{i-5}} & M_{{gyro}_{i-5}}\\ M_{{acc}_{i-10}} & M_{{gyro}_{i-10}}\\ \vdots & \vdots \\ M_{{acc}_{i-w}} & M_{{gyro}_{i-w}} \end{array}\right|,\;M_{acc}:acceleration\;of\;3\;axis;\;M_{gyro}:angular\;velocity\;of\;3\;axis;\;\;i:sampling\;time;\;w:size\;of\;window\;\left(1.8\;s\right);\;{Window}_{i}:Constructed\;window\;at\;i.$$

## 4. User Authentication System

**Figure 4:**
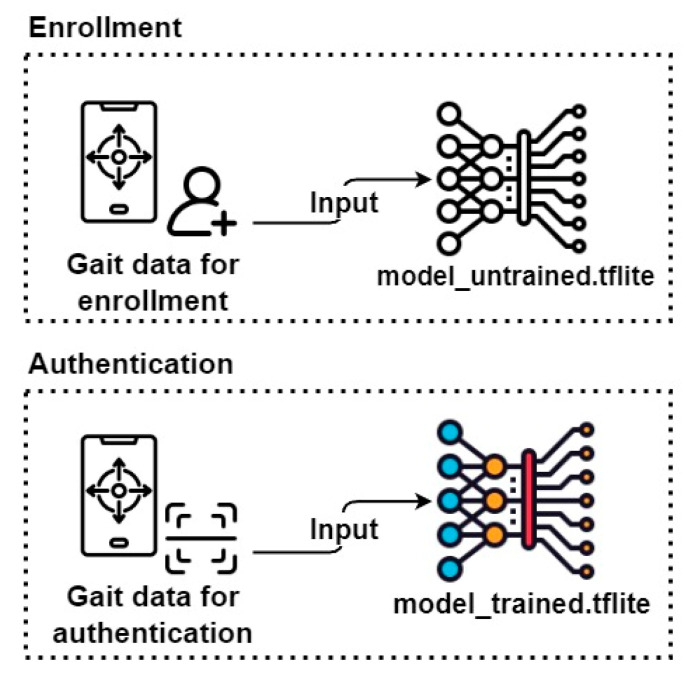
System overview.

Section 4 describes the deep learning model and interface used in our proposed authentication system. We developed a mobile application based on the Android operating system, registered the user’s gait data, and used a CNN-based deep learning model to determine whether a user was the legitimate smartphone owner. Gait data refer to the data generated from the accelerometer and gyroscope included in most smartphones when a person walks. Our mobile application, which registers and authenticates the user’s gait data, is defined as a user authentication system (hereafter referred to simply as a system). Figure 4 shows an overview of the system performing two main functions: “Enrollment” for registering the user’s gait data; and “Authentication” for confirming whether the user is legitimate. In the Enrollment process, the acceleration and angular velocity collected through the smartphone are entered into the model to register legitimate users’ gait data, and then the deep learning model is trained with the collected data. We call this data the training set. In the Authentication process, the newly collected acceleration and angular velocity are entered into the trained CNN to validate the users and grant them access to the system. We call this newly collected data the test set. The development environment of this study is described in Table 3.

### 4.1. Model Configuration

**Figure 5:**
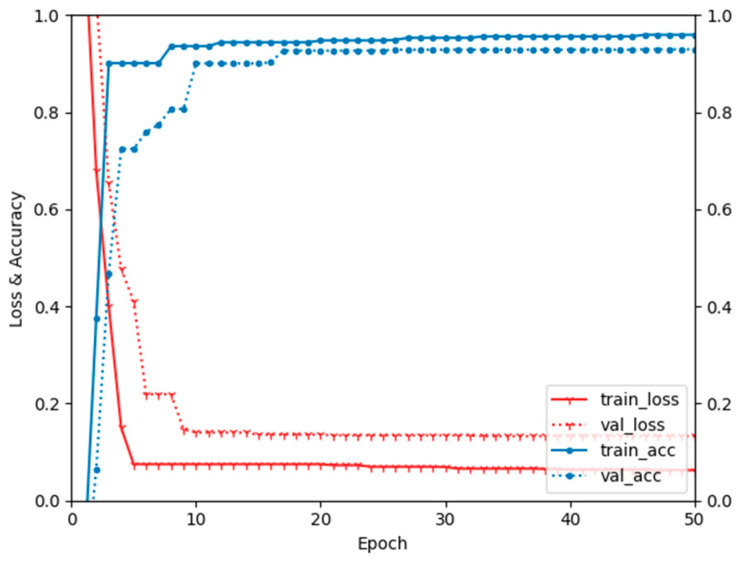
Loss and accuracy of on-device training.

A CNN model was constructed to implement the proposed personal authentication method. The input of the model is the gait data that have undergone the window processing described in Section 3, and the output is the probability of being a legitimate user. The configuration of the model is described in Table 4. After the initial configuration on the desktop, it was converted into a model that supports on-device training with TensorFlow Lite to be loaded into an Android mobile application. This conversion allows user authentication to be performed simultaneously with gait data training on the mobile device. We utilized Scikit-learn’s “train_test_split()” function to randomly divide the dataset into training and testing sets, with a split ratio of 6:4. This was achieved by specifying the parameters test_size (=0.4) and shuffle (=true). The CNN model’s configuration and the design of each neural network layer are outlined in Table 4. During model training, we employed “TensorFlow’s “ModelCheckpoint()” function and set the “save_best_only” parameter to true, ensuring that the model was saved only when its performance improved. Additionally, we allocated 10% of the test dataset for validation purposes. Figure 5 shows the loss and accuracy of on-device training and verification by epoch. It is evident from the graph that overfitting starts to occur around the 10th epoch. By examining the model’s accuracy calculations, we observed that the training set achieved an accuracy of approximately 95% in identifying legitimate, while the validation set achieved an accuracy of around 90%. Based on the information provided in Figure 5, the accuracy of the test set will be approximately 90%. To measure the training time, we conducted a total of 10 training runs, with an average duration of 12 min.

### 4.2. Model Generate and Mount to Device

**Figure 6:**
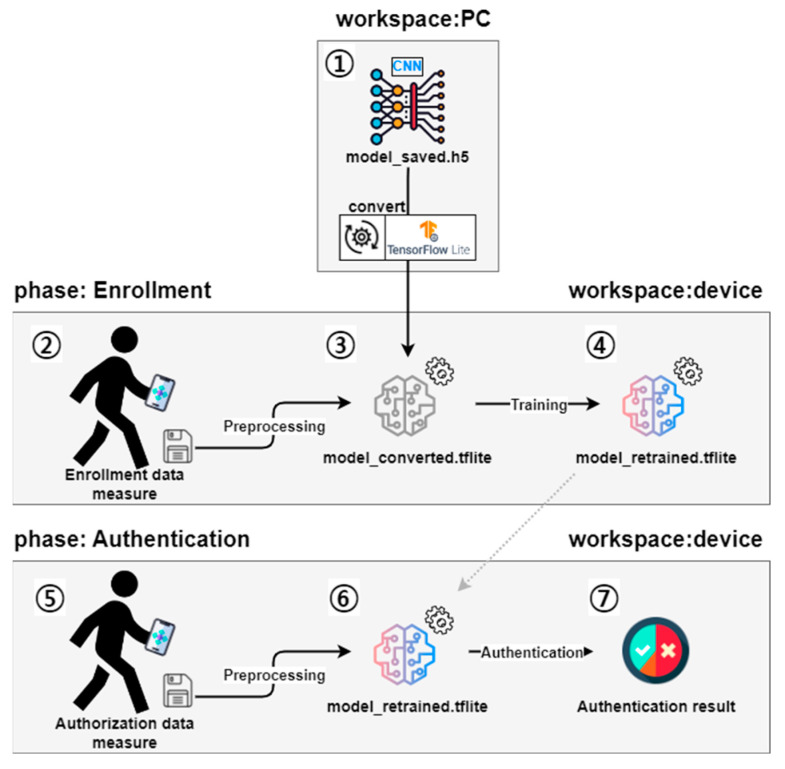
Model generation and mounting to mobile device.

The deep learning model constructed in this study should be embedded in a mobile device so that it can be trained and tested on the device. Figure 6 shows the process of training and testing the model on the device. First, participants configure the neural network and input data on their PCs then save the model that trained the data (“Model_saved.h5”). In order to save the model, it is necessary to go through the training process, but if random gait data is used to train the model, there is a possibility that the data will reduce the performance of the authentication function. Therefore, to prevent this problem, this study first trained dummy data with only the format of gait data and saved an empty model (“Model_saved.h5”). Then, we converted the saved model (“Model_saved.h5”) into a model for mobile devices (“Model_converted.tflite”) using the TensorFlow Lite library and mounted it on a smartphone. Second, we retrained “model_converted.tflite” with the gait data for enrollment collected in Section 3 to enroll the gait data of legitimate users. Finally, the third step is to input the gait data of the legitimate user to “model_retrained.tflite” to determine whether it is legitimate or not, and then check the result.

### 4.3. User Interface

**Figure 7:**
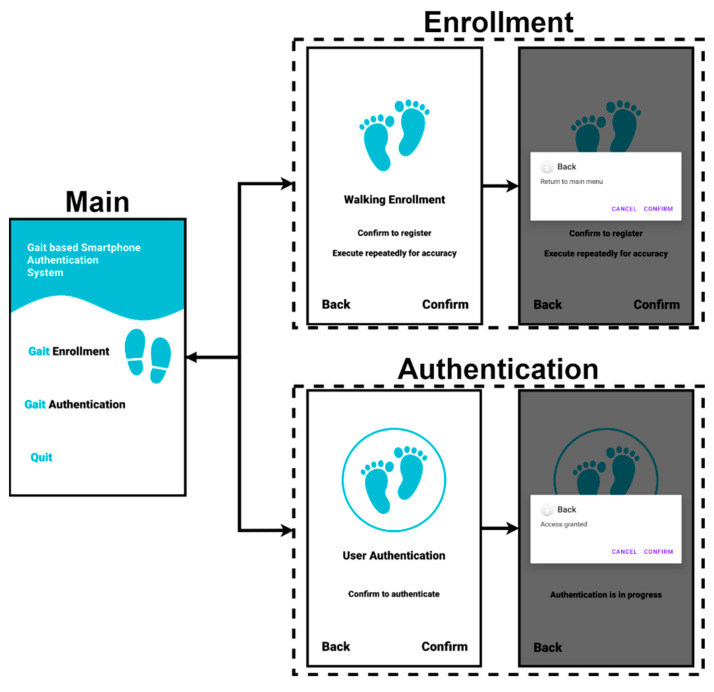
System interface.

The main user interface of the system with the CNN model is shown in Figure 5. As mentioned earlier, users can register their gait data collected in the Enrollment step in Figure 7 as legitimate user data and then check whether the newly collected gait data are legitimate in the Authentication step. These two functions can be selected on the main screen of the system first, and the process of registering the legitimate user’s gait data during Enrollment is required to use Authentication. First, a legitimate user selects the Enrollment step on the main screen and walks for approximately 10 s. The system stores the data collected for 10 s as the legitimate system user. A user who wants to use the system selects the Authentication menu on the main screen and walks for 1.8 s. The system determines whether the user is legitimate based on this data. If the user is determined to be a legitimate user, the system displays the result message “Access granted” on the screen.

## 5. Performance Results

In Section 5, we evaluate the system proposed in Section 4 using evaluation metrics. In this study, we used accuracy, precision, recall, and F1-score as metrics to evaluate the performance of the system, and the criteria used for each metric (TP, FN, FP, and TN) are shown in Table 5.

The authentication results of 10 users calculated by the above four evaluation metrics are shown in Table 6: the average accuracy was 0.917561; the average precision was 0.910181, average recall was 0.928293; and the average F1-score was 0.918055, all of which are above 90%. The closer the result of each metric is to 1, the higher the performance. The evaluation results of the system (average over 0.9) show that the proposed system has reliable performance. In our previous study [27], we needed to collect 7 s of gait data to authenticate a user, whereas with our improved system, we only need 1.8 s of gait data to determine whether a user is legitimate. Consequently, the performance improvement was nearly four-fold compared to the previous system. The proposed system confirms that gait data collected over a short period of time can be utilized for user authentication.

One of our study’s main results is demonstrating the robustness of the proposed authentication system to changes in the position of the smartphone. We collected data by placing smartphones in different positions and achieved an individual identification accuracy of over 90%, which indicates that the system can adequately reflect real-world environment where the position of the smartphone may vary. However, there are three notable limitations to note: First, the small number of participants (10) is insufficient for statistical validation. We need to recruit a larger sample size and additional participants to produce reliable results. Second, like previous research, our study was conducted in a controlled environment, i.e., a 20 m flat indoor floor. As a result, we acknowledge the limitation of not considering all the environmental factors typically taken into account, such as activity, age, and illness. In order to further improve the authentication system in practice, it is important to consider environmental factors that may affect walking patterns. In this study, we only considered the data collection position on the body; however, we should investigate the impact of other environmental factors, such as walking speed, ground type, age, and illness, on authentication accuracy. Finally, our system is not on par with state-of-the-art biometric methods such as fingerprint or iris recognition, which boast near-perfect accuracy. However, even these state-of-the-art biometric systems have limitations in certain situations, such as recognizing faces when wearing masks or glasses or detecting fingerprints when a foreign object is on the sensor. Therefore, we propose to incorporate our system as a secondary approach that can compensate for authentication errors while utilizing state-of-the-art biometrics as the primary approach.

## 6. Conclusions

In conclusion, this study addresses the environmental limitations of previous research on gait-based user authentication systems by developing a smartphone-based system using a CNN model. Previous studies were limited to collecting data in controlled environments that did not fully represent reality and to using data from a fixed position on the body for training the authentication system [9,10,11,12,13,14,15,16,17,18,19,20,21,22]. Because of these limitations, the potential exists for misidentification when an authentication system trained on data collected in one position on the body identifies data collected in another position. This study overcomes these limitations by collecting gait data from various positions on the body, allowing for flexibility in data collection that aligns more closely with real-world environments. The proposed authentication system demonstrates robust performance, achieving accuracy, precision, recall, and F1-score results above 90%. Compared to our previous study, the authentication time of the proposed model has been significantly reduced through the implementation of filtering techniques. This reduction signifies a four-fold improvement in performance, highlighting the efficiency of utilizing even a short duration of gait data for user authentication. The findings emphasize the potential of gait data as a secure and reliable means of authentication.

The system shows promise but requires further research and improvement. First, less-than-full consideration of environmental factors, e.g., failing to account for activity level and variations in floor conditions, hampers accuracy and reliability. Second, the small sample size of ten participants raises concerns about statistical validation and generalizability. Recruiting a larger and more diverse sample is necessary. Lastly, the system falls short compared to state-of-the-art biometric methods such as fingerprint or iris recognition. Addressing these limitations through research and development is crucial for enhancing performance, expanding applicability, and establishing secure and reliable user authentication. Incorporating environmental factors, larger samples, and complementary approaches to existing biometric methods will facilitate successful implementation.

The proposed system has practical applications such as alerting authenticated users in case of loss or theft and exploring age-based authentication possibilities. By training the system to distinguish between gait data from different age groups, it can provide an additional layer of security and customization. Furthermore, the system can be extended to incorporate additional biometric methods and enhance overall authentication accuracy.

In summary, this study contributes to the field of gait-based user authentication by developing a smartphone-based system that addresses previous limitations. The system’s robust performance, flexibility in data collection, and potential applications make it a promising approach to obtaining secure and reliable user authentication. Continued research and development in this area will further enhance the system’s performance and expand its practical applications in various domains, including security and healthcare.

## Figures and Tables

**Table 2:** Measure size and time of dataset.

ID	Device Position				Measure Time (s)
	Pocket _L_	Pocket _R_	Hand _L_	Hand _R_	
1	4605	4674	4533	4570	91.9
2	4395	4327	4033	3862	83.0
3	4897	4632	4419	4382	91.6
4	4746	4756	4830	4794	95.6
5	5501	5382	5207	5449	107.6
6	4854	4795	4416	4581	93.2
7	5297	5098	4709	4686	98.9
8	3837	4121	3900	4141	79.9
9	5585	5323	4931	4545	101.9
10	5036	4729	5142	5006	99.5

**Table 3:** Development environment.

Category	Description
Platform	Desktop (PC), smartphone (LM-G850N)
IDE	Vscode, Android Studio
Language	Python, Java, Kotlin
Package	Tensorflow 2.8.0, Keras, TensorFlow Lite
Category	Description

**Table 4:** Model configuration.

No	Layer (Type)
1	conv1d (Conv1D)
2	max_pooling1d (MaxPooling1D)
3	dropout (Dropout)
4	conv1d_1 (Conv1D)
5	max_pooling1d_1 (MaxPooling1D)
6	conv1d_2 (Conv1D)
7	max_pooling1d_2 (MaxPooling1D)
8	dropout_1 (Dropout)
9	conv1d_3 (Conv1D)
10	max_pooling1d_3 (MaxPooling1D)
11	flatten (Flatten)
12	dense (Dense)

Layers 1, 4, 6, and 9 (conv1d): The feature map is extracted via nesting using six filters of size 6; Layers 2, 5, 7, and 10 (max_pooling1d): The size of the feature map output from the conv1d layer is reduced, and the major features are emphasized and extracted through the maximum value (maxPool) of the feature map and passed to the next layer; Layers 3 and 8 (dropout): Overfitting is prevented in advance by setting the neurons in the hidden layer to 0 with a probability of 0.3; Layer 11 (Flatten): Data are converted into a one-dimensional list form.; Layer 12 (dense): As a network for classifying legitimate users, the softmax activation function is used to output a probability value between 0 and 1.

**Table 5:** Evaluation standards.

	Predicted Value	Legitimate User	Unregistered User
Actual Value			
Legitimate		TP	FN
Unregistered		FP	TN

TP: Legitimate users correctly classified as legitimate users; FN: Legitimate users incorrectly classified as unregistered users; FP: Unregistered users incorrectly classified as legitimate users; TN: Unregistered users correctly classified as unregistered users.

**Table 6:** Evaluation results.

ID	Accuracy	Precision	Recall	F1-Score
1	0.907	0.892	0.926	0.909
2	0.914	0.882	0.956	0.918
3	0.941	0.896	0.997	0.944
4	0.913	0.94	0.882	0.91
5	0.934	0.899	0.978	0.936
6	0.904	0.932	0.873	0.901
7	0.932	0.888	0.99	0.936
8	0.914	0.927	0.9	0.913
9	0.880	0.884	0.875	0.879
10	0.931	0.958	0.902	0.929

## References

[1] Abuhamad M., Abuhmed T., Mohaisen D., Nyang D. (2020). AUToSen: Deep-Learning-Based Implicit Continuous Authentication Using Smartphone Sensors. IEEE Internet Things J..

[2] Sudhakar S.R.V., Kayastha N., Sha K. (2021). ActID: An efficient framework for activity sensor based user identification. Comput. Secur..

[3] San-Segundo R., Echeverry-Correa J.D., Salamea-Palacios C., Lutfi S.L., Pardo J.M. (2017). I-vector analysis for Gait-based Person Identification using smartphone inertial signals. Pervasive Mob. Comput..

[4] Hutabarat Y., Owaki D., Hayashibe M. (2021). Recent Advances in Quantitative Gait Analysis Using Wearable Sensors: A Review. IEEE Sens. J..

[5] Connor P., Ross A. (2018). Biometric recognition by gait: A survey of modalities and features. Comput. Vis. Image Underst..

[6] Singh J.P., Jain S., Arora S., Singh U.P. (2018). Vision-Based Gait Recognition: A Survey. IEEE Access.

[7] Karampelas P., Bourlai T. (2018). A Survey of Using Biometrics for Smart Visual Surveillance: Gait Recognition. Surveillance in Action. Advanced Sciences and Technologies for Security Applications.

[8] Sprager S., Juric M.B. (2015). Inertial Sensor-Based Gait Recognition: A Review. Sensors.

[9] Leyva R., Santos G., Rocha A., Sanchez V., Li C.T. Accelerometer Dense Trajectories for Activity Recognition and People Identification. Proceedings of the 2019 7th International Workshop on Biometrics and Forensics (IWBF).

[10] Delgado-Escaño R., Castro F.M., Cózar J.R., Marín-Jiménez M.J., Guil N. (2019). An End-to-End Multi-Task and Fusion CNN for Inertial-Based Gait Recognition. IEEE Access.

[11] Adel O., Soliman M., Gomaa W. Inertial Gait-based Person Authentication Using Siamese Networks. Proceedings of the International Joint Conference on Neural Networks (IJCNN).

[12] Tran L., Hoang T., Nguyen T., Kim H., Choi D. (2021). Multi-Model Long Short-Term Memory Network for Gait Recognition Using Window-Based Data Segment. IEEE Access.

[13] Gadaleta M., Rossi M. (2018). IDNet: Smartphone-based gait recognition with convolutional neural networks. Pattern Recognit..

[14] Sun F., Mao C., Fan X., Li Y. (2019). Accelerometer-Based Speed-Adaptive Gait Authentication Method for Wearable IoT Devices. IEEE Internet Things J..

[15] Kothamachu A.R., Chakraborty B. Real Time Gait based Person Authentication using Deep Hybrid Network. Proceedings of the IEEE 4th International Conference on Knowledge Innovation and Invention (ICKII).

[16] Giorgi G., Saracino A., Martinelli F. (2021). Using recurrent neural networks for continuous authentication through gait analysis. Pattern Recognit. Lett..

[17] Ehatisham-ul-Haq M., Azam M.A., Naeem U., Amin Y., Loo J. (2018). Continuous authentication of smartphone users based on activity pattern recognition using passive mobile sensing. J. Netw. Comput. Appl..

[18] Kala N., Bhatia T., Aggarwal N. Person Identification and Characterization from Gait Using Smartphone. Proceedings of the 2019 11th International Conference on Communication Systems & Networks (COMSNETS).

[19] Xu W., Shen Y., Luo C., Li J., Li W., Zomaya A.Y. (2020). Gait-Watch: A Gait-based context-aware authentication system for smart watch via sparse coding. Ad Hoc Netw..

[20] Tsai Y., Hong Y.P. Center-Assisted Personal Gait Authentication Using Orientation Adversarial Feature Extraction. Proceedings of the 2019 IEEE 29th International Workshop on Machine Learning for Signal Processing (MLSP).

[21] Asuncion L.V.R., Mesa J.X.P.D., Juan P.K.H., Sayson N.T., Cruz A.R.D. Thigh Motion-Based Gait Analysis for Human Identification using Inertial Measurement Units (IMUs). Proceedings of the 2018 IEEE 10th International Conference on Humanoid, Nanotechnology, Information Technology, Communication and Control, Environment and Management (HNICEM).

[22] Choi J., Choi S., Kang T. (2022). Identification of Gait Patterns using Convolutional Neural Networks for Personal Authentication. J. Korean Inst. Inf. Technol. (JKIIT).

[23] He L., Ma C., Tu C., Zhang Y. Gait2Vec: Continuous Authentication of Smartphone Users Based on Gait Behavior. Proceedings of the 2022 IEEE 25th International Conference on Computer Supported Cooperative Work in Design (CSCWD).

[24] Watanabe Y., Kimura M. (2020). Gait identification and authentication using LSTM based on 3-axis accelerations of smartphone. Procedia Comput. Sci..

[25] Li Y., Hu H., Zhou G. (2019). Using Data Augmentation in Continuous Authentication on Smartphones. IEEE Internet Things J..

[26] Luca R., Bejinariu S., Costin H., Rotaru F. Inertial Data Based Learning Methods for Person Authentication. Proceedings of the 2021 International Symposium on Signals, Circuits and Systems (ISSCS).

[27] Choi J., Choi S., Kang T. (2022). Smartphone Authentication System using Personal Gaits and CNN. J. Korean Inst. Inf. Technol. (JKIIT).

